# Medicated corn feeders to disinfest cattle fever ticks, *Boophilus* (*Boophilus*) *microplus* (Acari: Ixodidae), from a suburban population of white-tailed deer, *Odocoileus virginianus* (Cervidae)

**DOI:** 10.1007/s10493-022-00699-7

**Published:** 2022-03-02

**Authors:** Donald B. Thomas, Roberta Duhaime

**Affiliations:** 1 Agricultural Research Service, Cattle Fever Tick Research Laboratory, USDA, 22675 North Moorefield Road, Edinburg, TX 77841 USA; 2 Animal and Plant Health Inspection Service, Cattle Fever Tick Eradication Program, USDA, 120 San Francisco Avenue, Laredo, TX 78040 USA

**Keywords:** *Rhipicephalus microplus*, *Boophilus*, Ivermectin, Cattle fever ticks, Eradication

## Abstract

Following its eradication from the USA, the cattle fever tick, *Rhipicephalus* (*Boophilus*) *microplus* (Canestrini), a vector of bovine babesiosis, has made episodic incursions into, and sometimes beyond, an established barrier zone separating tick-free from endemic areas. In large part the incursions involve hosting and transport by wild ungulates, particularly deer and antelope. One approach to disinfest ticks from wild hosts is with food baits medicated to stop parasites. The approach has had mixed success due to factors that have been previously identified with supplemental feeding of wildlife especially competition for the bait, social dominance behavior, and the availability of alternative food sources. Given that not all of the target hosts will intake a therapeutic dose of the medication (ivermectin) at all seasons of the year, an open question is whether the approach is efficacious as a stand-alone treatment or even as part of an integrated program. As detailed in the present study an intensive effort was successful in eradicating a local outbreak of fever ticks.

## Introduction

A recalcitrant outbreak of the southern cattle fever tick, *Rhipicephalus* (*Boophilus*) *microplus* (Canestrini) (Acari: Ixodidae) in the coastal counties of South Texas threatens the livestock industry in the USA as vectors of bovine babesiosis and bovine anaplasmosis (Tidwell et al. 2018). Unlike previous southern Texas incursions that are routinely extirpated by the Cattle Fever Tick Eradication Program, the coastal outbreak is centered on public land and sustained by wild ungulates (Lohmeyer et al. 2018). For example, in the particular case detailed herein, the cattle fever ticks were infesting a suburban population of white-tailed deer, *Odocoileus virginianus* Zimmerman (Artiodactyla: Cervidae). A road-killed deer at Port Mansfield, a coastal town in Willacy County, TX, was discovered to be infested with cattle fever ticks in May 2018. In situations like this when the deer occupy residential areas, wildlife management can be especially challenging. Because of restrictions on the use of firearms urban deer are not subject to hunting pressure and typically overpopulate. Mortality factors are mainly by collision with motor vehicles, domestic dog attacks, and malnutrition (Bowman 2011). On the aesthetic side, as charismatic wildlife, presence of the deer is considered desirable by many residents (i.e., those without gardens), but when the deer become carriers of ticks and tick-borne pathogens, the lack of a management plan is unsustainable.

Deer are not an optimal host for *Boophilus* ticks mainly because of their ability to groom themselves and one another (Cooksey et al. 1986). Nonetheless, wild deer can sustain a sylvatic population of cattle fever ticks (Kistner and Hayes 1970, Pound et al. 2010). The traditional use of acaricides to disinfest managed herds of cattle in pastures is not applicable to wildlife which cannot be gathered. Population models wherein infested deer intermingle with treated cattle predict considerable delays for achievement of disinfestation (Wang et al. 2016). Fever tick infested cattle are typically quarantined for a year or more under animal health regulations resulting in considerable cost to the producer and to the Cattle Fever Tick Eradication Program (Anderson et al. 2010).

Tick disinfestation techniques for wild deer consist of two practicable options: topical applications and medicated baits. An acaricide delivery system widely used in the eastern USA is the four-poster dust roller, a passive topical application device. Coupled with a corn-feeder, the rollers, dusted with pyrethroid, are mounted on either side of the feeder access such that the dust is self-applied when the deer’s head approaches the opening of the feeder (Pound et al. 2000). The limitation for the eradication program is that the southern Texas population of fever ticks has developed resistance to pyrethroids (Thomas et al. 2020). For example, Currie et al. (2020) found no correlation between permethrin treatment and infestation rate in their study of fever tick control on white-tailed deer in Zapata County, TX. The second option consists of medicating the corn bait with a systemic parasiticide, the macrocyclic lactone dewormer Ivermectin (Pound et al. 1996). Ivermectin does not kill the ticks outright, but rather inhibits the ability of the tick to imbibe blood (Jackson 1989). Without engorging on a blood meal, the tick is unable to reproduce. Pound et al. (1996) reported 100% efficacy based on zero larvae produced, when pasture confined deer were given 10 mg a.i. ivermectin in 0.45 kg corn per day. The issue for the eradication program is partly logistical; the ability to deliver an efficacious quantity of medicated corn to a substantive portion of the wild deer population.

An inverse correlation between serum ivermectin levels and tick infestation rate has been found for deer feeding at medicated corn bait stations (Currie et al. 2020). Thus, they concluded that ivermectin was a viable alternative to permethrin in a fever tick management program. Importantly, game cameras at the feeders documented overuse by adult males. If females and juveniles are systematically excluded by dominant adult male behavior such that they do not acquire a therapeutic dose of the medication, then the utility of the corn bait delivery system is compromised. The social interactions among deer, and application restrictions during the hunting season, reduces the efficacy of this approach (Currie et al. 2020). Given the need for treating the deer population at Port Mansfield and the few options available, the opportunity was taken to measure the effect of the medicated corn method under the conditions that therein existed. Within months of the detection of the infestation and implementation of the controls, the outbreak was contained and eliminated. The purpose of this report is to document the details of the application, the circumstances under which it was implemented, and the consequent results.

## Materials and methods

### The study area

Port Mansfield is located in Willacy County, TX, on the western coast of the Gulf of Mexico (at 26° 33′ 20″ N, − 97° 25′ 52″ W). The port occupies 14.8 km², with 563 dwellings. Approximately 9% of the port authority area is water due to a channel that serves the port. The topography is mostly level, occasionally inundated by storm surges. The habitat surrounding the town is saline coastal prairie dominated by sea oxeye daisy, *Borrichia frutescens* (L.) DC. and Carolina wolfberry, *Lycium carolinianum* Walter, with grasses, gulf cordgrass, *Spartina spartinae* (Trin.) Merr. and saltgrass *Distichlis spicata* (L.) Greene. Thorny tree cover increases with distance from shore and includes mesquite *Prosopis glandulosa* Torr. and Acacia, *Vachellia farnesiana* (L.) Wight & Arn. The largest trees, notably hackberry, *Celtis laevigata* Wildd., occur among the dwellings in the port village itself and the shade is often occupied by the deer in the daytime. On 24 February 2016, Port Mansfield became part of a broader surveillance zone because of detection of cattle fever ticks on adjacent ranch cattle herds. The port area is surrounded by fence; however, only on the north side was the fence effective as a barrier to wild game. Surveys of the area north of the game fence failed to detect fever ticks, thus Port Mansfield at the time of this study was the northern most infestation within the surveillance area. In contrast, the infestation rate on white-tailed deer taken and inspected during 2017 public hunts directly to the south in northern Cameron County, were at 55% positive for fever ticks (Olafson et al. 2018). Similarly, tick censuses on deer and nilgai in the 2018–2020 winter hunting months of November to February in Willacy and nearby Cameron counties were 10–20% infested with *R*. (*B*.)* microplus* (Osbrink et al. 2022).

### Tick counts

**Fig. 1:**
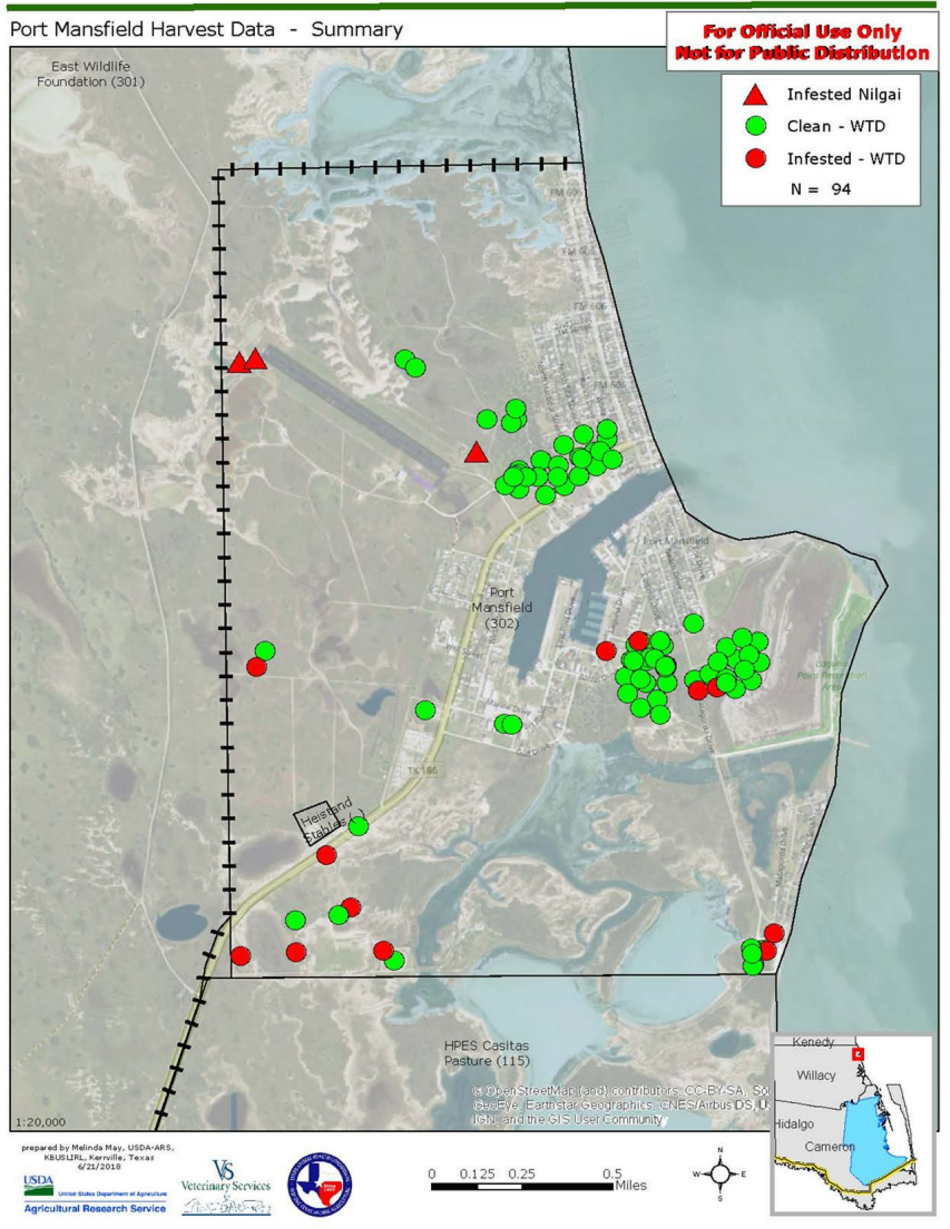
Map locations of culled white-tailed deer (*Odocoileus virginianus*) at Port Mansfield, TX, USA, in June 2018: infested (red dots) and non-infested (green dots) prior to implementation of medicated corn feeding. (Color figure online)

On successive occasions between April 2016 and February 2018, a total of 143 white-tailed deer were harvested from the Port area and examined for fever ticks. Harvesting was conducted by the Wildlife Services division of the United States Department of Agriculture (USDA) under permit from the Texas Department of Parks and Wildlife to the senior author. All deer and three nilgai antelope, *Boselaphus tragocamelus* (Pallas) (Artiodactyla: Bovidae), were free of fever ticks on those occasions. On 14 May 2018, a road-killed deer was found to be infested with fever ticks. Consequently, the Port Mansfield deer population was sampled serially between 17 May and 12 June to establish the rate of infestation (Fig. 1). An aerial survey (visual counts from rotary-wing aircraft at 30 mph speed, 200 m between sweeps) was conducted to determine the size of the deer population in the area, which was approximated at 400 head. Harvests of deer were conducted after dark by marksmen using night scopes and subsonic ammunition. The harvested deer were examined for ticks by Texas Animal Health Commission and USDA-Animal Plant Health Inspection Service (APHIS) inspectors. Tactile (scratching) examination of the pelt is considered reliable for adults and fully engorged nymphal stages. All ticks collected from individual deer were placed in labeled, plastic, screw-top bottles. The ticks were identified to species by a professional taxonomist and counted at the USDA-Agricultural Research Service (ARS) Cattle Fever Tick Research Laboratory, Edinburg, TX.

### Treatments

**Fig. 2:**
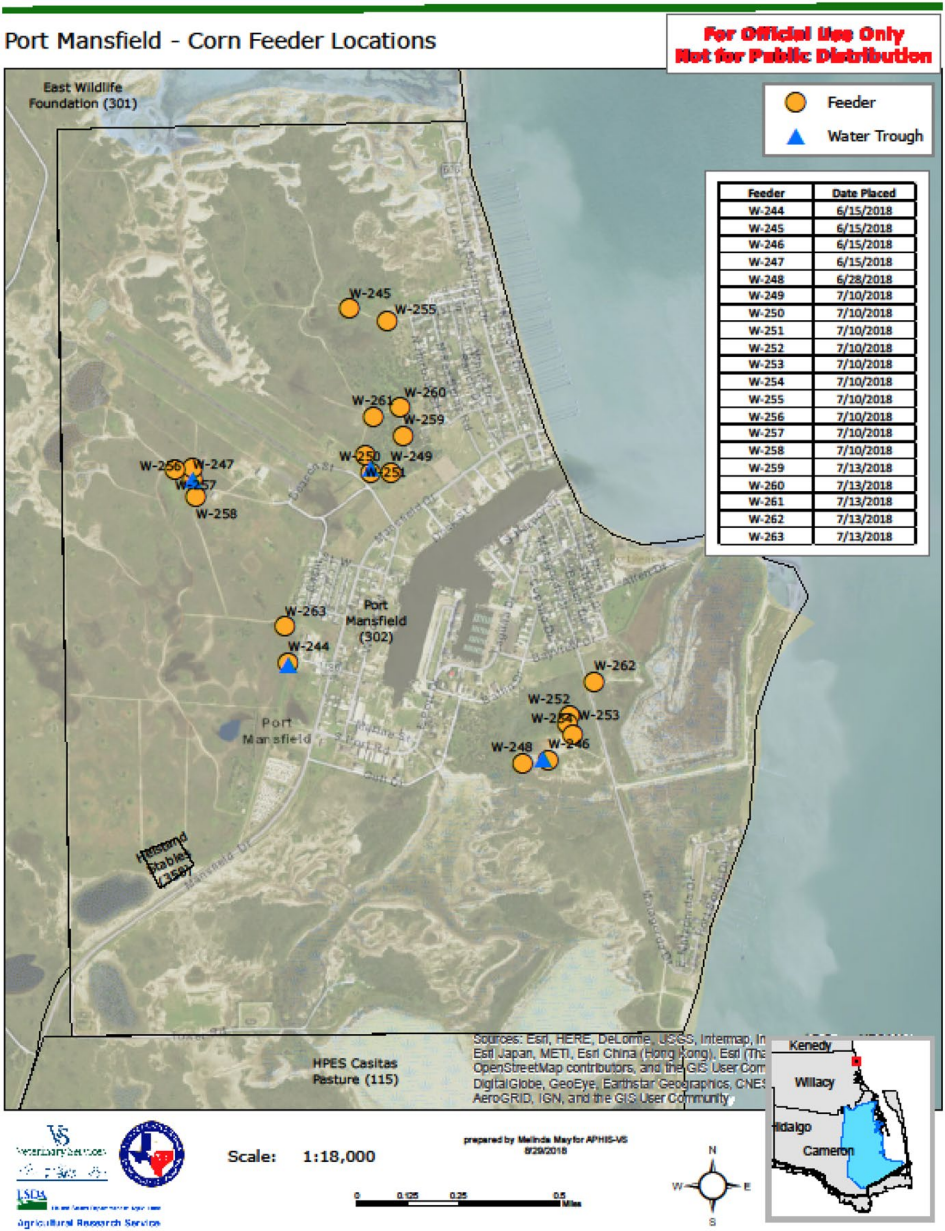
Medicated (ivermectin) corn feeder locations installed at Port Mansfield, TX, July 2018

Whole kernel corn was mixed with Ivomec® pour-on for cattle acquired from Merial Limited (Duluth, GA, USA). For dosing, 200 ml of the formulation containing 5 mg ivermectin/ml was mixed with 45 kg of clean corn to produce 10 mg of ivermectin active ingredient per 0.45 kg of corn (Pound et al. 1966). The treated corn was placed in gravity flow feeders which are commercially available, top-lid, plastic bins with four feed tubes, one at each corner (Texas Hunter Products, Meridian, MS, USA). Each feeder has a holding capacity of 140–160 kg of corn. A total of 20 feeders were installed between 15 June and 15 July 2018 within the Port Authority boundaries (Figure 2). The number of feeder sites was determined based on number and density of deer following cattle fever tick eradication program guidelines. Those are one feeder per 20–30 deer to minimize excessive competition and social dominance. Density of feeders is set so that deer do not have to travel more than 400–800 m (¼–½ mile) to access feed, equivalent to one feeder/50 ha to one feeder/200 ha (Bonilla 2017). Feed stations were enclosed with a 10-m-diameter welded wire hog-panel fence to exclude non-target animals (hogs, javelinas). The feeders were serviced and filled weekly with an approximate average of 4000 kg of medicated corn per month consumption rate.

### Ivermectin residues

On the nights of 13–15 November 2018, after 5 months of medicated corn treatment, 81 deer were harvested at the site. The gender and age of each animal was recorded with age determined by tooth wear (Cain and Wallace 2003). From each animal a sample of adipose tissue was collected from the tail base and/or from organ fat. If adequate adipose tissue was not available, a piece of kidney was collected. Tissues were placed in a whirl-pak and kept on ice until shipped for analysis to the Texas Medical Veterinary Diagnostic Laboratory in College Station, TX. Ivermectin residues were quantified with LC-mass spectrometry (Danaher et al. 2006) with detection limit at 1.0 ng/ml (= ppb) and quantifiable limit at 10 ng/ml. Due to its high lipophilic nature, ivermectin tends to sequester in the adipose tissue. As the fat is metabolized the ivermectin is eliminated through the liver, and thus highest levels are found in the fat and liver (Gonzalez-Canga et al. 2009). Kidneys are also routinely included for macrocyclic lactone residue testing (Danaher et al. 2006). However, in malnourished animals concentration is higher and clearance is slower from the fat and thus a poorer measure of intake or therapeutic levels compared to serum or plasma.

### Statistical analysis

Least squares regression analysis was used to correlate serum ivermectin levels with adipose tissue levels after √x-transformation of the raw data, using the online quickcalcs linear regression calculator (Graphpad.com). Means and standard deviations were also calculated and compared with a non-paired t-test with the same on-line calculator.

## Results

### Tick counts

Soon after the index case was discovered in May 2018 the deer population was censused for infestation rate. Including the index case, 13% (12/91) of the deer were found infested with fever ticks (Table 1). Additionally, three nilgai antelope were also inspected, and unlike those previously found uninfested, all three were positive for cattle fever ticks. The timing of the census in late-May, early-June coincided with the typical spring-time peak in fever tick populations. Within 4 weeks the 20 feeder stations were installed and functioning. In November, after 5 months of operation, the deer population was recensused. Over three consecutive nights, 81 deer were harvested, and all found to be free of fever ticks (Table 1). Medicated corn feeders remained in operation through the fall of 2019 at which time the supplements were withdrawn. Withdrawal of the medication 60 days prior to the hunting season allows consumption of the harvested deer. In January 2020, 101 adult deer were harvested as part of a supervised youth hunt at Port Mansfield. All harvested deer were inspected and all were found negative for cattle fever ticks. In November-December 2021, 60 adult deer were harvested over 3 weekends under the same circumstances as the prior year and all were free of cattle fever ticks.

**Table 1:** Percentage of culled white-tailed deer (*Odocoileus virginianus*) infested with fever ticks, *Rhipicephalus* (*Boophilus*) *microplus*, by date at Port Mansfield, TX

Date	No. culled deer	No. infested	% infested
April 2016–Feb 2018	143	0	0
May 14, 2018	1	1	100
May 17, 2018	18	3	16.7
May 21, 2018	15	2	13.3
May 22, 2018	23	2	8.7
May 23, 2018	7	1	14.3
June 11, 2018	20	3	15.0
June 12, 2018	7	0	0
Nov 13, 2018	31	0	0
Nov 14, 2018	19	0	0
Nov 15, 2018	31	0	0

### Tissue samples

Of the 81 harvested animals, 64% had detectable levels of ivermectin in either their serum or tissues with notable variation due to age and gender (Table 2). A greater proportion of adults were ivermectin positive at 69% compared to juveniles at 55%, which conforms with the study by Currie et al. (2020). Also, a greater proportion of males were ivermectin positive at 72% compared to females at 59%, also mirroring the study by Currie et al. (2020). Hence, 79% of adult males were ivermectin positive, whereas in comparison only 36% of juvenile females were positive. Of the 44 animals that were seropositive, five had lower than therapeutic levels (8 ppm). The serum-positive levels ranged from 0.1 to 114 ppb. Levels in tissue and serum were very different. Seropositive adults had a mean of 32.3 ± 25.6 ppb whereas the tissues had a mean of 863.0 ± 756.3 ppb (range: 0–2428 ppb). There was, however, a significant correlation between serum and tissue levels (r^2^ = 0.612, *F* = 45.6, *P* = 0.0001). Almost all of the positive tissues were adipose tissue. Of 33 animals for which kidney samples were taken because of insufficient fat tissue, 25 were negative both for the tissue and the serum. Five of the kidney samples had low amounts (9–24 ppm) and those animals were also seronegative (< 0.1 ppb). In three animals the kidney samples were high for ivermectin (569–742 ppb) and their serum was correspondingly high (17–33 ppb).

**Table 2:** Mean (± SD) percentage of culled white-tailed deer (*Odocoileus virginianus*) positive for ivermectin in either or both serum and tissue by gender and age class at Port Mansfield, TX

Class	No. harvested deer	% ivermectin positive	Serum (ppb)	Tissue (ppb)
Adult males	14	78.6	25.9 ± 31.6	1041 ± 948
Adult females	38	65.8	18.3 ± 23.4	438.7 ± 655.2
All adults	52	69.2	20.3 ± 26.1	600.8 ± 791.7
Juvenile males	18	66.7	4.38 ± 10.1	170.0 ± 394.0
Juvenile females	11	36.4	0.04 ± 0.06	2.54 ± 5.41
All juveniles	29	55.2	2.74 ± 8.25	105.9 ± 320.6
Totals	81	64.2	14.4 ± 23.1	423.7 ± 703.8

Mean concentrations of ivermectin by age class in ng/ml (= ppb). For means of ivermectin-positive individuals only, see text

## Discussion

Deer need to consume approximately 1% of their body weight per day to reach therapeutic serum levels of ivermectin, > 8 ng/ml (Pound 2010). The daily intake dose of the deer is approximately 0.22 mg/kg assuming a 45 kg deer eats 0.45 kg of corn per day. A feeding rate of 0.22 mg/kg should produce maximum blood serum levels of approximately 30 ppb (Pound et al. 1996). The target concentration of 30 ppb assures a high degree of efficacy even in those deer that may consume as little as one-third of the targeted dosage. Serum levels of just 10 ppb (one-third of the dosage) should produce 99.9% efficacy against ticks feeding on treated animals (Nolan et al. 1985; Miller et al. 1989; Pound et al. 1996). The mode of action of ivermectin is relevant. Muscle paralysis interferes with blood sucking and ultimately the ability to engorge. A tick that cannot engorge cannot reproduce. Thus, efficacy is measured by reduced fecundity, rather than mortality. As serum levels decline below the therapeutic level, the tick fecundity increases in linear fashion (Davey et al. 2010). The adult females harvested post-treatment (N = 38) had a mean of 18.3 ± 23.4 ppb ivermectin in their serum compared to a mean of 25.9 ± 31.6 ppb in the adult males (N = 14). The difference was not statistically significant (*t* = 0.94, *df* = 50, *P* = 0.35) and the means were well above the therapeutic dosage. However, the difference between adults and juveniles was much greater and significant (*t* = 3.52, *df* = 79, *P* < 0.001); adults had a mean of 20.3 ± 26.1 ppb, whereas for juveniles the mean was 2.74 ± 8.25 ppb, averaging below therapeutic levels. These results strongly suggest that malnourished animals, as indicated by the absence of body fat, were those not taking advantage of the corn feeders and, hence, were (with few exceptions) below therapeutic levels for ivermectin. Six seropositive animals had no detectable residues of ivermectin in their tissues. In deer, as in other ruminants, orally ingested ivermectin reaches peak serum concentration within 24 h (MacIntosh et al. 1985; Gonzalez-Canga et al. 2009). Hence, it is likely that these animals had only recently accessed the medicated corn.

Because at the time of analysis none of the animals were infested with fever ticks the correlation between infestation levels and serum or tissue levels could not be determined. The serum half-life for orally administered ivermectin in cattle is 15.1 days with 90% of elimination through the feces (Gonzalez-Canga et al. 2009) and is detectable in the fat for 28 days post-treatment in deer (Danaher et al. 2006). Hence, animals without detectable levels of ivermectin at the time of examination, may have consumed the medication but their systems had cleared prior to analysis. The 64% positive rate measured in this study thus represents a snapshot of the population. Whereas it is possible that a different set of individuals would be found to be seropositive a month or more prior to the sample date, it is unlikely that the persistent association with gender and age would be much different.

Corn feeders have been used as a deer management tool for decades. To study deer feeder use patterns, Bartoskevitz et al. (2003) marked pelleted feed so that it could be detected in hunter-harvested deer. Depending on season and habitat they found 25–50% of the males had used the feeders but only 0–25% of the females. Season is a factor because of increased aggression during the mating (antler) season (October–December) when mature males exhibit aggressive behavior (chasing) against yearlings and young. By contrast, during the non-mating season grooming between adults and yearlings is common (Hirth 1977). In the study at Port Mansfield only 64% of the population was ivermectin positive, whereas in the study by Currie et al. (2020) 78% were ivermectin positive—this difference may be partly explained because the Port Mansfield blood sampling was in November, the middle of the mating season, whereas the Zapata blood sampling was in February–April. Interestingly, Currie et al. (2020) also reported that fawns had low ivermectin levels, perhaps because they were not competitive at the feeders. Fawns were not sampled in the present study as fawns are not present at the time of year when we sampled (Cook et al. 1971) although it is worth noting that fawns can get medicated through their mother inasmuch as ivermectin is sequestered in the milk (Cerkvenik-Flajs and Grabner 2002). Another factor may be population density. Donohue et al. (2013) found that at all densities, does avoided bucks at feeders, but as population density increased social pressures to avoid feeders also increased. On South Texas ranches, typical deer density is one deer for every 10–15 ha (Webb et al. 2007). The density at Port Mansfield was four deer for every 1 ha.

Although no ticks were found on any of the deer in the three post-treatment culls there is a reasonable chance that complete eradication was not achieved because not all of the deer were examined. The absence of ticks in the first cull especially has to be considered in light of the practical matter that only the engorged nymphs and adults are detectable in a scratch inspection. Also, early in the infestation cycle the peak in adults tends to be episodic because of synchronization of the life cycle with the P1 case, which was likely a single infested animal that moved into the premises dropping female ticks. Over multiple generations (approx. 6 weeks ea), the synchronization fades as cohorts of off-host questing larvae are spread over time, often for several months. Still, by the time of the second post-treatment cull with about half of the deer examined, there would be a roughly 50% chance that an infestation rate of 1% would be undetected. On the other hand, an infestation rate of 5% or more is highly unlikely (> 99%) to have been undetected. One can only conclude that the treatment significantly reduced and perhaps eradicated the population under the circumstances that existed at the time.

The issue for managers is what proportion of the host population needs to have efficacious levels of acaracide to cause the target pest population to decline and ultimately die out. In this case near or actual eradication was successful through rapid implementation of the corn feeders upon detection of the infestation by the monitoring program. The infestation rate in this herd soon after it was first detected was approx. 14%. In contrast, during the Zapata County study reported by Currie et al. (2020), a chronic infestation prevailed with around 67% of the deer infested by fever ticks. Other factors that undoubtedly contributed to the apparent eradication, was the absence of alternative hosts (especially cattle), and reduction in the population of deer to about half of pretreatment numbers. Another consideration is that the normal withdrawal of treatment prior to the hunting season was in this case reversed and the treatments continued through that season and into the next, keeping the pressure on the tick population.
